# Independent Component Analysis and Source Localization on Mobile EEG Data Can Identify Increased Levels of Acute Stress

**DOI:** 10.3389/fnhum.2017.00310

**Published:** 2017-06-16

**Authors:** Bryan R. Schlink, Steven M. Peterson, W. D. Hairston, Peter König, Scott E. Kerick, Daniel P. Ferris

**Affiliations:** ^1^Human Neuromechanics Laboratory, School of Kinesiology, University of Michigan, Ann ArborMI, United States; ^2^Human Research and Engineering Directorate, United States Army Research Laboratory, Aberdeen Proving GroundMD, United States; ^3^Institute of Cognitive Science, University of OsnabrückOsnabrück, Germany; ^4^University Medical Center Hamburg-EppendorfHamburg, Germany

**Keywords:** EEG, stress, ICA, shooting, source localization, prefrontal cortex

## Abstract

Mobile electroencephalography (EEG) is a very useful tool to investigate the physiological basis of cognition under real-world conditions. However, as we move experimentation into less-constrained environments, the influence of state changes increases. The influence of stress on cortical activity and cognition is an important example. Monitoring of modulation of cortical activity by EEG measurements is a promising tool for assessing acute stress. In this study, we test this hypothesis and combine EEG with independent component analysis and source localization to identify cortical differences between a control condition and a stressful condition. Subjects performed a stationary shooting task using an airsoft rifle with and without the threat of an experimenter firing a different airsoft rifle in their direction. We observed significantly higher skin conductance responses and salivary cortisol levels (*p* < 0.05 for both) during the stressful conditions, indicating that we had successfully induced an adequate level of acute stress. We located independent components in five regions throughout the cortex, most notably in the dorsolateral prefrontal cortex, a region previously shown to be affected by increased levels of stress. This area showed a significant decrease in spectral power in the theta and alpha bands less than a second after the subjects pulled the trigger. Overall, our results suggest that EEG with independent component analysis and source localization has the potential of monitoring acute stress in real-world environments.

## Introduction

The brain is a dynamic and complex structure, and knowledge of the underlying cognitive processes is crucial to develop better treatment and rehabilitation methods for neurological disorders. Moreover, it is important to analyze these processes in real-world situations because it gives a more accurate measurement of what is truly occurring than in laboratory studies. To this end, mobile brain imaging, via electroencephalography (EEG), has become an effective tool for measuring cortical activity in freely moving humans (Gwin et al., 2011; Kline et al., 2014; Seeber et al., 2014; Nathan and Contreras-Vidal, 2015; Bradford et al., 2016; Oliveira et al., 2016). Mobile brain imaging has several potential benefits, including the diagnosis and treatment of different neurological disorders under real-world conditions (Lau et al., 2012). However, real-world environments present a new set of challenges, such as maladaptive effects on cognition (McDowell et al., 2013).

One effect that is consistently seen when individuals are subjected to real-world conditions is stress. Stress occurs as a state change while an individual responds to internal or external events. The onset of a stressful encounter activates the hypothalamus-pituitary-adrenal gland axis (HPA axis), a specialized part of the neuroendocrine system that leads to the secretion of cortisol into the blood stream (Smith and Vale, 2006). Modulation of this neuroendocrine response affects several parts of the body, including cardiovascular function and the immune response. More importantly for research involving mobile brain dynamics, stress can affect an individual’s cognition and motor performance (Tsigos and Chrousos, 2002).

Acute stress has been shown to affect several areas of the cerebral cortex, the most notable and well-researched being the prefrontal cortex. Mild acute stress can cause a reduction in prefrontal cortex functioning, which in turn can have large effects on our behavior and our ability to respond to changes in our environment (Arnsten, 2009). The dorsolateral prefrontal cortex’s working memory ability is also decreased when an individual is stressed, potentially due to the increased levels of cortisol (Qin et al., 2009). Gärtner et al. (2014) showed that theta band synchronization decreased when participants were subject to acute stressors. Chronic stress can even lead to remodeling of the prefrontal cortex structure (McEwen et al., 2016), an important consideration for longitudinal studies and studies involving state-trait interactions. Stress affects other areas of the brain as well, including the insular cortex, which has been correlated with sympathetic nerve activity (Wang et al., 2016). The hippocampus and amygdala have also been shown to be affected by stress-response pathways (McEwen et al., 2016). Therefore, if we are to analyze brain dynamics in real-world conditions, we must also have reliable methods to measure the effects of stress, especially those in the prefrontal cortex.

There are several types of direct and indirect approaches that have been used to monitor acute and chronic stress. Perhaps the most effective way of quantifying the response of the HPA axis would be to measure the hypothalamus directly, but this is not possible with current neurophysiological techniques. Magnetic resonance imaging (MRI) provides the only useful depiction of the hypothalamus (Seidenwurm, 2008), but MRI is not conducive for measuring brain dynamics during real-world situations and interactions. Therefore, traditional measures have attempted to investigate stress by looking at the downstream effects of the HPA axis. This is primarily done by measuring the level of cortisol in blood, urine or saliva. Blood serum cortisol is the most direct approach and has very good temporal resolution, but it requires intravenous access. Urinary cortisol can also be quite invasive in terms of data collection, and renal conditions can have a large effect on the level of cortisol (Hellhammer et al., 2009). Both of these methods may induce iatrogenic stress due to the nature of obtaining the sample (Bozovic et al., 2013). In contrast, obtaining salivary cortisol measurements is less invasive and easier to obtain, although its temporal resolution is not as strong and often has very noisy results when compared to blood cortisol. For these reasons, it is difficult to consider salivary cortisol as the gold standard for stress measurement (Hellhammer et al., 2009). As a result, the need exists to develop a non-invasive method with good temporal resolution and few side effects for the subject.

The benefits of using EEG as a stress monitor include fine temporal resolution, mobility, and the ability to record data for extended periods of time. Several studies have previously explored the potential of EEG as a biomarker for acute stress (Hamid et al., 2010; Sulaiman et al., 2011; Putman et al., 2014). Hamid et al. (2010) found that high subjective stress scores correlated with lower right-to-left hemispheric ratios in the alpha and beta band power. Putman et al. (2014) found that the theta/beta power ratio negatively correlated with self-reported trait attentional control. Sulaiman et al. (2011) took the power spectrum from subjects during an IQ test and analyzed it using a relative energy ratio, Shannon entropy, and spectral centroids. However, these studies have all focused on EEG data in sensor space, and two of them used subjective measures to determine if stress was induced in their participants. They did not employ source localization, and none have delivered conclusive results that indicate that a method for monitoring acute stress has been established. To our knowledge, no studies have investigated the use of independent component analysis with source localization as a stress monitor. Based on our previous work using independent component analysis and source localization to quantify electrocortical dynamics in humans during movement, we wanted to determine if the same methods could detect differences in a functional human motor task that had varying level of induced stress.

The purpose of this study was to determine if high-density EEG with independent component analysis and source localization could quantify differences in a shooting task between a control condition and a condition with increased acute stress. Healthy, young, novice subjects shot at a target with an airsoft rifle with and without the threat of being shot at by an experimenter with a second airsoft rifle. We measured three physiological parameters (salivary cortisol, heart rate, and skin conductance) to assess stress levels in the subjects to compare to the EEG data. Previous research has shown that salivary cortisol and skin conductance readings can be indicative of stress levels (Christie, 1981; Hellhammer et al., 2009; Poh et al., 2010; Reinhardt et al., 2012) and that prefrontal cortical activation is correlated with stress levels (Arnsten and Goldman-Rakic, 1998; Qin et al., 2009; Arnsten, 2015), especially prefrontal theta band spectral power (Gärtner et al., 2014, 2015). Based on the reasoning above, we expected a participation of prefrontal cortex structures. However, the contribution of further cortical areas and the frequency bands involved forms an exploratory component of the study. We hypothesized that: (1) the stress condition would have greater physiological indicators of stress compared to the control condition or baseline condition, and (2) the stress condition would have reduced synchronization in prefrontal cortex electrocortical spectral power compared to the control condition.

## Materials and Methods

### Subjects

Eleven healthy volunteers, all male, between the ages of 19–30 years participated in the study. None had any history of major lower limb injury or known neurological or locomotor deficits. Additionally, all subjects were right-hand dominant. All study procedures were approved by the University of Michigan Internal Review Board, and all subjects provided informed, written consent before participating in accordance with the Declaration of Helsinki.

### Protocol

**FIGURE 1:**
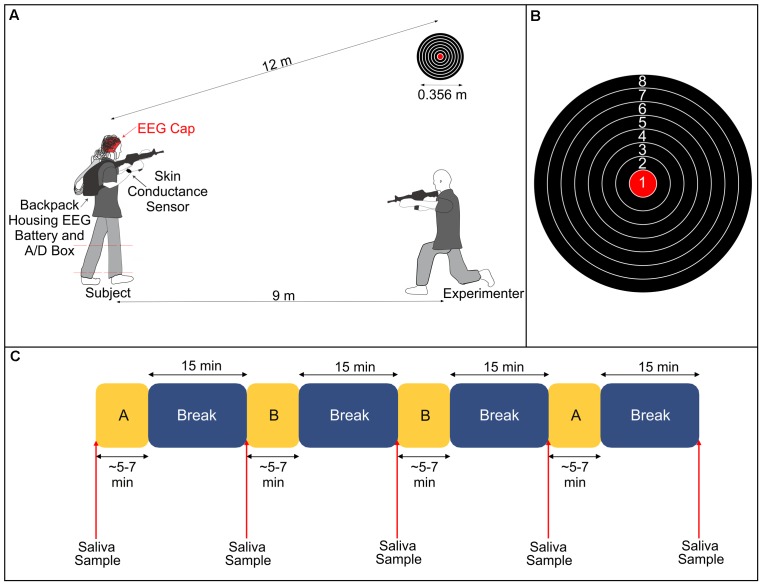
**(A)** Illustration of experimental setup. The subject fired an airsoft rifle at a stationary target 12 m away during all conditions. During the Stress condition, an experimenter, positioned 9 m away and at a 30° angle from the target, fired a different airsoft rifle at the subject’s lower legs (dashed red lines denote the region that the subject was hit). **(B)** Diagram of the target and the numerical value associated with each gradation. **(C)** Breakdown of the experimental protocol. Subjects completed two blocks of each condition (A and B, representing either Stress or Control), with each block followed by a 15-min rest period. We took saliva samples immediately before starting the experiment and at the conclusion of each break. The lengths of A and B varied depending on the subjects’ self-selected paces.

Subjects performed a stationary shooting task with an airsoft rifle that shoots plastic pellets in our laboratory (**Figure 1**). Subjects fired 50 shots per condition at a target with a diameter of 0.356 m positioned approximately 12 m away from where they stood. The target had eight gradations, and subjects were instructed to try to hit the bullseye with each shot. Prior to beginning the experimental conditions, an experimenter instructed the subject how to hold, aim and fire the rifle. The subjects were then allowed to fire at the target from the same distance as during the experiment until they said they felt comfortable using the rifle. The experiment consisted of two conditions (Control and Stress) that were each repeated twice (henceforth referred to as Control 1, Control 2, Stress 1, and Stress 2). The order of the experiment followed an A-B-B-A pattern, where A and B represent the two different conditions. The selection of which condition (Control or Stress) was represented by ‘A’ or ‘B’ was randomized for each subject to avoid any possible order effects.

In the Control condition, we instructed subjects to aim the rifle at the target and fire one shot at the target. They were then instructed to bring the rifle down out of its aimed position and rest for a brief moment. They repeated this procedure until 50 shots had been fired. At the conclusion of 50 shots, subjects sat quietly for 15 min. In the Stress condition, subjects performed the exact same protocol as the Control condition. However, throughout the duration of the trial, an experimenter used a second airsoft rifle to fire shots in the direction of the subject. The experimenter was positioned approximately 9 m away from the subject at a horizontal angle of 30° from the target. We informed subjects prior to the start of the experiment that, during the Stress condition, the experimenter would fire the same number of shots as them (50) and that they could potentially be hit on either of their lower legs (between the ankle and the knee) at any point throughout the trial. Subjects were also informed that they could be hit as many as 50 times or as little as 0 times. In actuality, subjects were only hit a total of five times combined across both Stress trials. We instructed the subjects to focus on their task of shooting at the target and to not look over at the experimenter who was shooting at them. The experimenter remained in an aimed position throughout the trial so that there would be no indication as to when they would fire at the subject.

### Quantitative Measurements

We recorded scalp EEG data using a 128-channel Biosemi ActiveTwo system (Biosemi, Amsterdam, The Netherlands). The ActiveTwo system allows for additional external electrodes to be recorded simultaneously. We placed four of these electrodes on the neck to measure the subject’s neck muscle activity at the following locations: left and right splenius capitis muscles and left and right levator scapulae muscles. Two other electrodes were placed on the subject’s chest in order to measure electrocardiography. One was placed on left side of the subject’s chest, and the other was placed directly over the sternum. The less noisy of the two electrodes was used to calculate the subject’s heart rate.

We recorded skin conductance from each of the subject’s wrists using E3 wristbands (Empatica, Milan, Italy) (Poh et al., 2010). The wristbands were attached to the subject before any other equipment was applied and continuously recorded data from the beginning of the EEG preparation until the conclusion of the entire experiment. Additionally, we collected saliva samples from each subject at six different time points: (1) Immediately upon arriving and completing the informed consent document; (2) At the conclusion of subject preparation, just before the first trial was to begin; and (3–6) 15 min after each of the four trials. The first two samples were averaged as a baseline sample. Saliva samples were collected using oral swabs (Salimetrics, State College, PA, United States) that the subject kept underneath their tongue for 2 min. The samples were analyzed using a Salimetrics ELISA kit to quantify the concentration of salivary cortisol in each of the samples (Strazdins et al., 2005). We scheduled all experiments for the afternoon and ensured that subjects had been awake for at least 5 h to avoid the typical cortisol increase in response to waking up in the morning (Wust et al., 2000).

We placed a switch behind the triggers of both the subject’s and the experimenter’s rifles in order to record the exact time at which the shot was fired. Additionally, a second experimenter kept track of the five shots that hit the subject and noted them by pressing a button immediately after the subject was hit. This served as an indication that the signal we received from the experimenter’s trigger switch just before the button was pressed was a shot that hit the subject. Finally, we recorded which ring on the target that the subject hit with each shot in every trial using a high-speed digital camera.

### Data Analysis

Raw skin conductance data were analyzed in Ledalab^1^ and deconvolution was performed using the program’s default parameters (Benedek and Kaernbach, 2010a,b). Skin conductance response (SCR) list values were computed using a threshold of 0.05 μS for all conditions (Schmidt and Walach, 2000). Heart rate data (as measured from one of the two Biosemi external electrodes, chosen based on which had the largest, cleanest signal) were first high pass filtered at 1Hz. We then ran Cleanline^2^ on the filtered data and applied a bandpass filter with cutoffs of 5 and 20 Hz using filtfilt in Matlab (The MathWorks, Inc., Natick, MA, United States). We then imported filtered data into Kubios (Tarvainen et al., 2014), ran an auto QRS-peak detection pipeline, and manually checked the results for accuracy, adjusting any incorrect peak detections. Using these results, we calculated heart rate, heart rate variability [defined as the standard deviation of the R–R intervals (Malik, 1996)], and the ratio of high to low frequency electrocardiogram power.

Electroencephalography data were processed using custom scripts in EEGLAB (Delorme and Makeig, 2004) according to the procedure used by Kline et al. (2014). We removed channels using established factors (magnitude, kurtosis, correlation, and standard deviation) according to Gwin et al. (2010). However, when evaluating kurtosis, we excluded channels greater than five standard deviations. We identified and rejected noisy frames, or time periods of EEG data exhibiting high power across all channels (greater than six times the interquartile range of the channels). We also removed a small sample of additional channels that were evenly spread across the head in order to ensure we had convergence of our independent component analysis. Overall, the channel rejection resulted in an average rejection of 38.4 channels per subject (range, 37–41; standard deviation, 1.3), which is comparable to other recent mobile EEG studies (Bradford et al., 2016; Oliveira et al., 2016).

We re-referenced the remaining channels to an average reference. We applied adaptive mixture independent component analysis [AMICA (Palmer et al., 2006, 2008)] on the cleaned data sets to transform the EEG channel data into temporally independent component signals (Makeig et al., 1996). We used the DIPFIT function in EEGLAB (Oostenveld and Oostendorp, 2002) to model each independent component as an equivalent current dipole within a boundary element head model based on the MNI (Montreal Neurological Institute, Quebec, Canada) brain. We removed components from further analysis if the best-fit dipole accounted for less than 85% of the scalp map variance (Gwin et al., 2011), or if the scalp map or spectra indicated that they were the result of muscle or eye artifact (Jung et al., 2000a,b). We epoched the data from -2 to 2 s centered around the time at which the subject pulled the trigger. We created additional epochs with the same time span that were centered around the time the experimenter pulled the trigger during the Stress condition. We clustered independent components across all 11 subjects based on similarities in scalp topography, spectra, and dipole location using a *k*-means clustering algorithm available in EEGLAB (Ehinger et al., 2014). We found five brain clusters based on 1/f power spectrum that contained components from more than half of the subjects (≥6): dorsolateral prefrontal cortex (7 independent components, 6 subjects), somatosensory association complex (15 independent components, 8 subjects), left sensorimotor area (10 independent components, 7 subjects), pre-motor and supplementary motor cortex (10 independent components, 8 subjects), midcingulate (16 independent components, 6 subjects). These five clusters were used for further analyses. We used a Wilcoxon (rank-sum) test to analyze mean power differences between Control and Stress conditions within a 2 Hz frequency window (α = 0.05) (Bradford et al., 2016).

We made an event-locked plot of spectral power change from baseline (defined as the average over the entire epoch) around each stimulus during the target-shooting task for each of our clusters. The stimulus was defined as when the subject pulled the trigger as measured by the button behind the trigger. We computed the power spectrum for each independent component for every stimulus. We averaged the power spectrum from each condition (Control and Stress) over all stimuli for each component and over all components for each cluster. Therefore, both conditions used the same spectral baseline. To allow spectral changes over time to be easily visualized, we subtracted the baseline spectral power. Our intention was to focus on acute stress, so we were not interested in the effects of stress on baseline EEG activity. These plots, showing spectral change from baseline, are referred to as event-related spectral perturbations (ERSPs; Makeig, 1993; Gwin et al., 2011). For the ERSP plots, time zero represents the time at which the trigger was pulled. We used standard bootstrapping methods in EEGLAB (Delorme and Makeig, 2004) to determine the regions that had a significant difference from baseline. We bootstrapped from the entire epoch and set all non-significant ERSP values (*p* > 0.05) compared to this distribution equal to zero. The remaining non-zero values represent power (in dB).

### Statistical Analysis

We analyzed all skin conductance data, heart rate data, salivary cortisol data, shooting scores, and shot times using a general linear model for repeated measures (ANOVA) in SPSS 22 (IBM SPSS Statistics for Windows, Version 22.0, Armonk, NY, United States). We reported the statistics with a significance level set at *p* < 0.05. We then ran *post hoc t*-tests on these variables to check for pairwise significance between the different conditions (*p* < 0.05). ERSP data were analyzed using bootstrapping methods in EEGLAB with a significance level set at *p* < 0.05.

## Results

### Physiological Measurements

**FIGURE 2:**
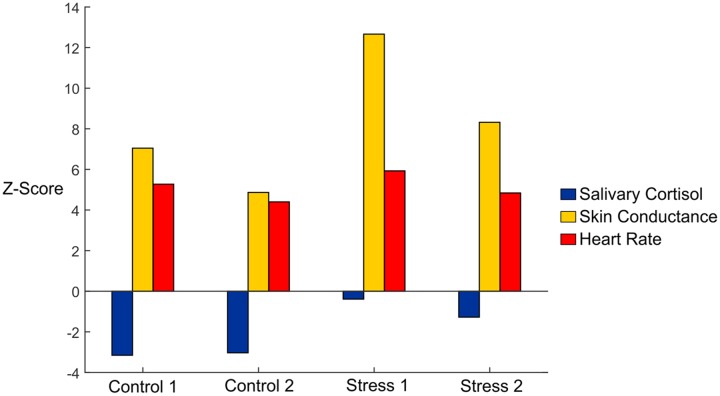
*Z*-scores of salivary cortisol (blue), skin conductance (gold), and heart rate (red) data in each of the experimental conditions. Data are shown with mean baseline values subtracted for each variable, and the *Z*-scores were calculated using the standard error for each variable.

First, we used conventional measures, including skin conductance, salivary cortisol, and heart rate data, to verify stress induction (**Table 1**). We observed an overall significance effect for skin conductance (*F*_4,40_ = 6.657, *p* < 0.05, η = 0.400). To better visualize the effects of each condition vs. Baseline, we plotted *Z*-scores of the salivary cortisol, skin conductance, and heart rate in **Figure 2**. We normalized our variables to Baseline by subtracting the mean of each variable from the Baseline condition and then dividing by the standard error of the Baseline condition. Skin conductance responses were the highest for subjects during both of the Stress trials, where subjects averaged 23.66 and 16.61 counts/minute for Stresses 1 and 2, respectively (**Figure 2**, gold bars). The skin conductance from Stress 1 was nearly eight times the magnitude we observed from the baseline condition, a statistically significant difference, and a significant difference was also observed relative to the Control 2 condition (*p* < 0.05 for both). Stress 2 was also significantly higher than the Baseline condition (*p* < 0.05), although it should be noted that one subject was determined to be a non-responder, defined as an individual who exhibits less than 0.002 μS/min on average throughout the experiment (Simons et al., 1983).

**Table 1:** Physiological parameters [skin conductance, salivary cortisol, heart rate, heart rate variability, and the ratio of high frequency (HF) to low frequency (LF) band power] for all subjects during Baseline, Control conditions, and Stress conditions.

	Baseline	Control 1	Control 2	Stress 1	Stress 2
Skin Conductance (counts/min)	3.11 ± 5.38^b,d,e^	14.55 ± 15.99^a^	11.01 ± 16.22^d^	23.66 ± 16.23^a,c,e^	16.61 ± 13.52^a,d^
Salivary Cortisol (μg/dL)	0.22 ± 0.04^b,d^	0.16 ± 0.03^a,d^	0.16 ± 0.04^a,d^	0.21 ± 0.05^b,c^	0.19 ± 0.11
Heart Rate (beats/min)	68.55 ± 11.39^b,c,d,e^	86.67 ± 16.55^a,c^	83.68 ± 15.80^a,b,d^	88.92 ± 18.36^a,c,e^	85.18 ± 15.69^a,d^
Heart Rate Variability (ms)	0.076 ± 0.023	0.084 ± 0.032	0.078 ± 0.034	0.092 ± 0.055	0.081 ± 0.039
HF/LF Power	8.51 ± 3.93	2.84 ± 2.39^a^	1.95 ± 1.80^a^	2.78 ± 3.23^a^	2.22 ± 2.19^a^


*^a^Denotes a statistical significance with Baseline at the 0.05 level. ^b^Denotes a statistical significance with Control 1 at the 0.05 level. ^c^Denotes a statistical significance with Stress 1 at the 0.05 level. ^d^Denotes a statistical significance with Stress 1 at the 0.05 level. ^e^Denotes a statistical significance with Stress 2 at the 0.05 level. Data are shown as mean ± standard deviation.*

Subjects exhibited higher salivary cortisol levels in both of the Stress trials compared to the Control trials (**Figure 2**, blue bars). We observed an overall significance (*F*_1.895,18.95_ = 3.596, *p* < 0.05, η = 0.264) and found significant differences between Stress 1 and both Control trials (*p* < 0.05). The Baseline cortisol level was also significantly higher than both Control trials (*p* < 0.05) and compared very closely to the values found in the Stress trials. This indicates that our subjects may have had some initial stress/anxiety when arriving at the lab and going through setup but were able to relax as the experiment began.

Our subjects had an average resting heart rate of 68.55 beats/min, and this jumped to 88.92 beats/min in the first Stress condition. Our ANOVA results showed an overall significance (*F*_2.28,22.79_ = 28.432, *p* < 0.05, η = 0.740), and we saw statistical significance between Stress 1 and the Baseline and Control 2 heart rate values (*p* < 0.05 for both). We also investigated heart rate variability, which was calculated as the standard deviation of the RR intervals (**Figure 2**, orange bars). No significant differences were seen between any conditions for heart rate variability (*F*_2.283,22.28_ = 0.745, *p* = 0.502, η = 0.069). Finally, we looked at the ratio of high frequency power to low frequency power for each of the conditions. We saw an overall significance among the conditions (*F*_4,40_ = 4.384, *p* < 0.05, η = 0.305). The baseline condition was found to be significantly lower compared to all experimental conditions (*p* < 0.05 for all), but no other significant results were observed. Overall, the strong responses we saw with the skin conductance, salivary cortisol, and average heart rate measurements confirmed that the experimental paradigm reliably and repeatedly elicited an acute physiological stress response.

### Task Performance

Subjects performed reasonably well at the shooting task regardless of whether or not they were at risk of being shot at by the experimenter. The highest (worst) average score occurred during Stress 1 (Mean: 2.85 ± 0.52), although it was only 0.32 higher than the lowest (best) score, which occurred during Control 2 (2.53 ± 0.51). We did not observe any statistical significance (*F*_3,30_ = 1.4, *p* = 0.262, η = 0.123). We also analyzed the time subjects took in between shots for all four trials. We found that subjects shot at the target faster during the Stress conditions (Stress 2: 6.16 ± 1.63 s between shots; Stress 1: 6.40 ± 1.37 s) than during the Control conditions (Control 1: 7.67 ± 2.70 s; Control 2: 6.58 ± 1.89 s). There was an overall significance from the ANOVA (*F*_2.221,6.644_ = 6.339, *p* < 0.05, η = 0.388), and the time between shots was significantly lower for Stresses 1 and 2 when compared to Control 1 (*p* < 0.05 for both), indicating that subjects tried to complete the task faster when under stress.

### EEG Results

**FIGURE 3:**
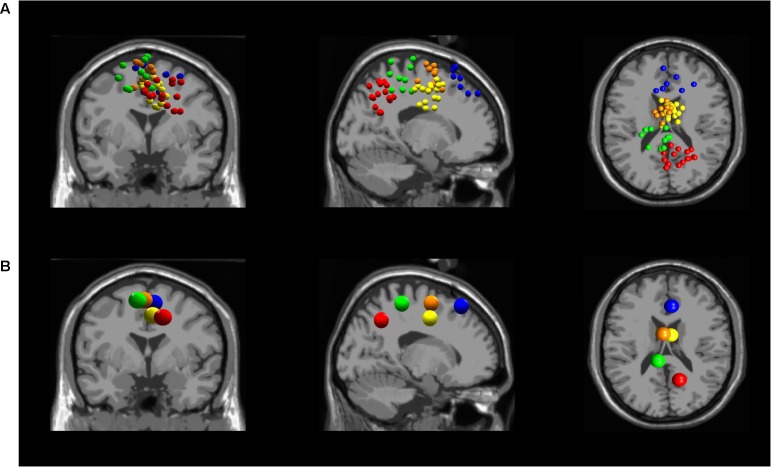
**(A)** All independent component locations for each of the five averaged independent components in the (from left to right) coronal, sagittal, and transverse planes. Five clusters were found in different locations in the brain: Dorsolateral Prefrontal Cortex (blue), Somatosensory Association Complex (red), Left Sensorimotor Area (green), Pre-motor and Supplementary Motor Cortex (orange), and Midcingulate (yellow). **(B)** Independent cluster centroids plotted in the (from left to right) coronal, sagittal, and transverse planes.

**FIGURE 4:**
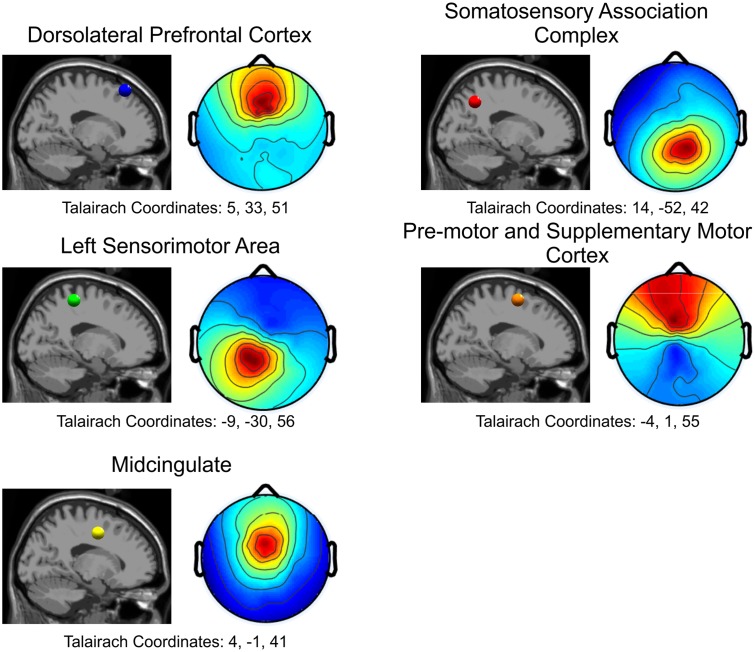
Cortical locations for all five independent components in the sagittal plane and the corresponding topographical maps. Blue: Dorsolateral Prefrontal Cortex; Red: Somatosensory Association Complex; Green: Left Sensorimotor Area; Orange: Pre-motor and Supplementary Motor Cortex; Yellow: Midcingulate.

Five independent component clusters met our criteria of having dipoles with greater than 85% of the scalp map variance and occurrence in at least 6 of our subjects (**Figure 3**). We located clusters in the dorsolateral prefrontal cortex, somatosensory association complex, left sensorimotor area, pre-motor and supplementary motor cortex, and the midcingulate (**Figure 4**).

### Event-Related Spectral Perturbations

**FIGURE 5:**
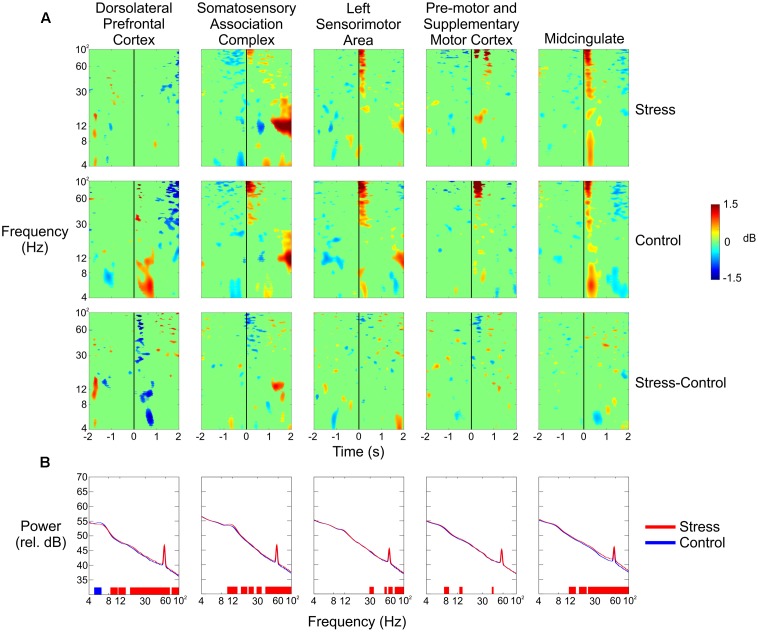
**(A)** Event-related spectral perturbation plots for (from top to bottom) the Stress condition, the Control condition, and the difference of the two for all five independent components (columns). The vertical black line indicates the time the subject pulled the trigger. **(B)** Spectral power of each of the five independent components in the Stress (red) and Control (blue) conditions. Blue vertical lines at the bottom of each plot indicate that the Control condition had significantly greater power in that region; red lines indicate that the Stress condition had significantly greater power (*p* < 0.05).

We observed large changes in spectral power in the theta and alpha bands for the dorsolateral prefrontal cortex (**Figure 5A**, top row). During the Control conditions (combined), there was a significant amount of synchronization from 4 to 12 Hz between 250 and 750 ms following the trigger pull that was not evident during the Stress conditions. We also saw a small amount of desynchronization 1 s before the trigger pull in the theta band during the Control condition that did not occur during the Stress condition.

A significant increase in spectral power could be seen in the alpha band for the somatosensory association complex 1–2 s after the subject pulled the trigger for the Stress condition vs. the Control condition (**Figure 5A**, second column). Very small spectral changes were seen for both clusters from the pre-motor and supplementary motor cortex, with the most notable occurring as a decrease in theta power 1 s before the trigger pull and then an increase 1 s after (**Figure 5A**, third column). In the anterior cingulate, there was a noticeable decrease in theta power half a second after the trigger pull (**Figure 5A**, fifth column).

**Figure 5B** shows the spectral power for each of the five clusters in the Control and Stress conditions (blue line and red line, respectively) for the entire duration of each condition. With the exception of the lower theta range, the prefrontal cortex had significantly higher power in the Stress condition throughout nearly the entire frequency range.

**FIGURE 6:**
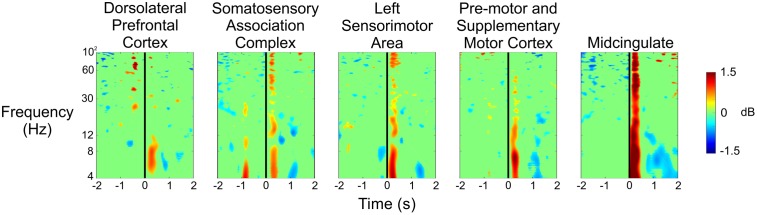
Event-related spectral perturbation plots showing the changes in power associated with the experimenter’s trigger pull (vertical black line) during the Stress condition for all five independent components.

**Figure 6** shows the spectral power for each of the five clusters at the time the experimenter pulled the trigger while shooting at the subject during the Stress condition. We saw significant increases in spectral power immediately after the experimenter’s trigger pull, especially for the theta and alpha bands in each of the five clusters. Additionally, we observed desynchronizations in spectral power in the theta band approximately 1–2 s after the experimenter’s trigger pull in the pre-motor and supplementary motor cortex, as well as the midcingulate.

**FIGURE 7:**
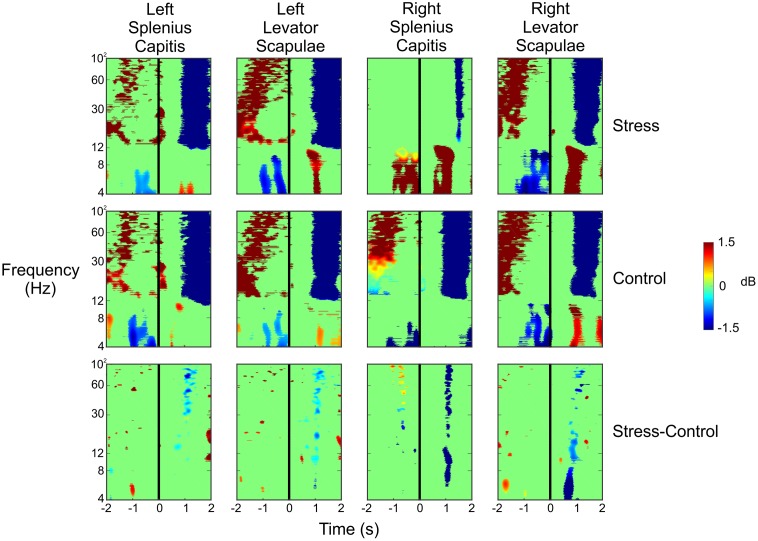
Event-related spectral perturbation plots for the neck muscle activity during the Stress and Control conditions, as well as the difference of the two conditions. The vertical black line indicates the time the subject pulled the trigger.

To ensure that the data resulted from cortical activity and not muscle activity (potentially as the subject flinches or tenses up as shots are fired in their direction), we analyzed the ERSPs for the four electrodes placed on the subjects’ necks. We placed an electrode at each of the following four locations: left splenius capitis, left levator scapulae, right splenius capitis, and right levator scapulae. **Figure 7** shows the ERSPs for each of these four electrodes in the same time window as the ERSPs shown in **Figure 5**. For both the Stress and Control conditions, we saw large increases in spectral power for the beta and gamma bands approximately 1–2 s before the subject pulled the trigger for each of the four muscles. This was followed by a very large desynchronization in the beta and gamma bands 1 s after the trigger pull, likely indicating the point at which the subjects began to relax and lower the rifle. However, these large changes in spectral power do not match what we see in each of the clusters presented in **Figure 5**, indicating that the neck muscles are not influencing our source activity.

**FIGURE 8:**
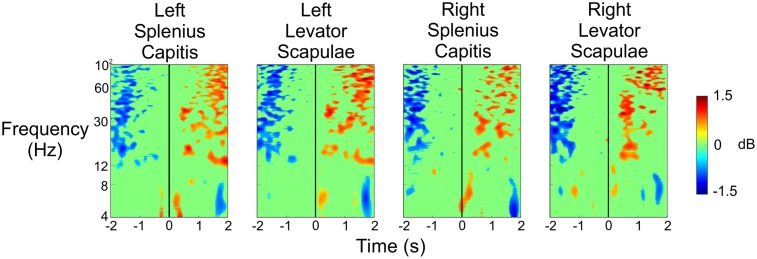
Event-related spectral perturbation plots for the neck muscle activity during the Stress condition when the experimenter shot at the subject. The vertical black line indicates the time at which the experimenter fired a shot (including both hits and misses) at the subject.

**Figure 8** shows the results of the same analysis for these neck electrodes when the subject was being shot at so that the results from **Figure 6** could be validated. We noticed a strong desynchronization in the beta and gamma bands 1–2 s before the subjects were shot at, and then we saw an increase in spectral power in these same frequency bands 1–2 s after the shot was fired. There were also some small increases in spectral power in the theta and lower alpha bands just after the shot was fired. Once again, we did not observe any spectral changes that would account for the strong activity we saw in **Figure 6** immediately after the shot was fired, indicating that the resulting activity in the different brain regions was not due to a sudden, large increase in muscle activity.

## Discussion

Based on significantly higher skin conductance responses and salivary cortisol levels observed in the Stress conditions, being shot at with an airsoft rifle during a shooting task was sufficient to increase stress levels in young novice subjects, similar to the findings in other related studies (Nieuwenhuys and Oudejans, 2010; Taverniers and De Boeck, 2014). Our three main measures of stress, skin conductance, salivary cortisol, and heart rate, all demonstrated some significant differences in elevated levels when being shot at compared to the control conditions. Our subjects did not have a significant decrease in shooting accuracy in the Stress condition compared to the Control condition, in contrast to Nieuwenhuys and Oudejans (2010). However, we did observe that our subjects shot faster during the Stress conditions, which agrees with their results and suggests that subjects may have been shooting faster to reduce the amount of time they are at risk of being hit. In this prior work, Nieuwenhuys and Oudejans (2010) studied police officers’ shooting accuracy with and without the presence of a threatening opponent, finding decreased accuracy in the threat/stress conditions. The differences in stress-induced accuracy between our study and Nieuwenhuys and Oudejans (2010) may be related to prior shooting experience or profession of the subjects, but the differences in protocols (airsoft vs. soap pellets, hit success rates) may also have influenced the outcomes. Additionally, their research paradigm involved reactive shooting and required perceptual discrimination and decision making, whereas our study was self-paced without any decision-making elements. Therefore, the strong skin conductance, salivary cortisol, and heart rate responses indicate that the paradigm we chose was highly efficient at inducing reliable levels of acute stress.

The EEG analysis supported the hypothesis that prefrontal cortex theta synchronization should be reduced in the Stress condition compared to the Control condition. For the dorsolateral prefrontal cluster, there was reduced spectral power in the theta band and alpha band after firing at the target. However, there were few differences in prefrontal cortex spectral activity in the 2 s prior to firing at the target. This suggests that when subjects were primarily focused on the motor task of shooting at the target, there was less of a discernible difference in prefrontal activity between the Stress and Control conditions. After shooting the rifle, when the subjects were not primarily focused on the motor task, a reduction in theta prefrontal spectral power was evident. During this time, subjects were likely focused only on the threat of being shot, and this reduction in theta power may be the result. We observed similar differences in alpha spectral power as well. The finding of decreased theta synchronization matched results from previous studies examining purely cognitive tasks that used visual scenes of graphic violence to induce stress in human subjects (Gärtner et al., 2014, 2015). In those studies, the Stress condition had reduced prefrontal spectral synchronization in the theta band compared to the Control condition. Frontal theta synchronization is associated with decreased anxiety (Mizuki et al., 1992), so this decrease in frontal theta activity suggests that our subjects felt more anxiety during the Stress condition than during the Control condition. The prefrontal cortex has long been known to be associated with memory, perception and monitoring of diverse cognitive processes (Siddiqui et al., 2008). Specifically, the dorsolateral prefrontal cortex focuses on implementation of control and adjusting behavior in response to changes in task demands (MacDonald et al., 2000). However, when an individual encounters a stressful situation, the prefrontal cortex can become impaired, causing diminished decision making abilities and error processing (Arnsten, 2009), as well as a reduction in working memory (Qin et al., 2009). The prefrontal spectral power results from our study suggest that EEG can be used to provide an electrocortical indicator of added stress, but that the effect is dependent on motor behavior. When our subjects were focusing on the shooting task, there were no substantive differences in prefrontal theta or alpha spectral power between stress and control conditions. It was only after the subjects fired the shot the differences in spectral power emerged.

The decrease in the alpha activity during the Stress condition in the dorsolateral prefrontal cortex is consistent with previous findings. Kerick et al. (2007) also found an increase in ERSP alpha synchronization when marksmen began performing a stationary shooting task, but they saw less of an increase when a dual-task scenario was introduced (arithmetic, recognizing friendly vs. non-friendly targets). A decrease in alpha activity may be indicative of perceptual or judgment demands. We may be seeing a similar trend due to the fact that our subjects were likely devoting some of their attention and focus onto preparing to be shot at during the Stress condition.

Several studies have previously explored the potential of using EEG data as a biomarker of stress. Haak et al. (2009) looked at the frequency of eye blinks in EEG data while subjects controlled a car on a computer screen in a virtual environment, as well as the overall regions of the brain that were active when their subjects performed a math exercise. They found a connection between eye blink frequency and perceived level of stress, although they couldn’t conclude that any specific areas were connected to events in their experiment. Putman et al. (2014) found a strong correlation between the EEG theta/beta ratio and an individual’s level of vulnerability to the effects of cognitive performance anxiety. They found that subjects with elevated ratios showed stronger declines of subjectively experienced attentional control. Although these studies are good indications that brain activity may contain information that reveals an acute level of stress, none of these previous studies confirmed they had induced acute stress in their subjects using objective measurements (e.g., cortisol, electrodermal activity, etc.). These studies all relied on questionnaires to determine their subjects’ levels of stress. Therefore, while their results may be promising, it may not be valid to conclude that they were actually examining stress in their subjects. Furthermore, none of these studies looked at EEG during motion.

Although we were able to observe some interesting findings, our study has some limitations. First, we did not inquire about any specific events (e.g., midterm exams, relationship problems, etc.) that may have affected our subjects’ levels of daily stress when they participated. It is therefore possible that some of our participants were experiencing stress from other aspects of their life while they took part in the experiment, which may have had effects on our objective measurements. Second, it is possible that some of our subjects may have adapted to the stressors as the experiment progressed, thereby washing out any stressful effects in the latter portions of the procedure. From casual conversation with our subjects, we did not get the sense that this was the case, but this was a subjective assessment and does not necessarily reflect the true effect.

This work has several benefits that future research could examine. While our analyses focused on naïve, inexperienced subjects, it would be useful to additionally test experienced shooters to see if the brain dynamics change. Haufler et al. (2000) found an increase in the upper alpha (10–11 Hz) power in the left hemisphere for marksmen when compared to novice shooters, a result that suggests a more efficient motor pattern when firing their weapon. They also found that novice shooters had reduced theta activity compared to experienced shooters. It is also possible that our task was too challenging for naïve subjects and therefore washed out any potential differences in performance. Furthermore, it would be interesting to see how training naïve subjects affects their levels of stress. Oudejans (2008) investigated the effects of reality-based practice with handgun shooting for police officers. They found that their subjects performed worse initially when presented with live opponents instead of cardboard targets, but after training sessions, there was no deterioration in shooting performance between the two conditions. Gevins et al. (1997) observed that subjects who practiced both verbal and spatial tasks increased frontal theta activity while alpha activity decreased. Since we observed decreases in alpha activity when subjects were under acute levels of stress, it would be important to see if we would get an even stronger desynchronization after subjects became more proficient at their task.

In summary, we have shown that independent component analysis and brain source localization has the potential to be a monitor of acute stress in humans through the identification of specific clusters in the brain, namely in the dorsolateral prefrontal cortex. While previous researchers have explored EEG as a means to monitor acute levels of stress, none have shown conclusive results that point toward a potential cortical biomarker. Future work should be aimed at looking at these effects in larger subject populations, as well as the effects that training may have.

## Author Contributions

BS, SP, WH, and DF designed the experiment. BS and SP performed the experiments. BS, SP, WH, PK, SK, and DF analyzed and interpreted the data. BS, SP, and DF drafted the manuscript and all authors approved the final version.

## Conflict of Interest Statement

The authors declare that the research was conducted in the absence of any commercial or financial relationships that could be construed as a potential conflict of interest.

## Footnotes

1 www.ledalab.de

2 https://www.nitrc.org/projects/cleanline/
